# A Hybrid Mayfly-Aquila Optimization Algorithm Based Energy-Efficient Clustering Routing Protocol for Wireless Sensor Networks

**DOI:** 10.3390/s22176405

**Published:** 2022-08-25

**Authors:** Gobi Natesan, Srinivas Konda, Rocío Pérez de Prado, Marcin Wozniak

**Affiliations:** 1 Department of Computer Science and Engineering, Dr. Mahalingam College of Engineering and Technology, Pollachi 642003, Tamilnadu, India; 2 Department of Data Science, CMR Technical Campus, Hyderabad 501401, Telangana, India; 3 Telecommunication Engineering Department, University of Jaén, 23700 Linares, Spain; 4 Faculty of Applied Mathematics, Silesian University of Technology, 44-100 Gliwice, Poland

**Keywords:** Aquila optimization algorithm, cluster head, mayfly, routing protocol, wireless sensor networks

## Abstract

In recent times, Wireless Sensor Networks (WSNs) are becoming more and more popular and are making significant advances in wireless communication thanks to low-cost and low-power sensors. However, since WSN nodes are battery-powered, they lose all of their autonomy after a certain time. This energy restriction impacts the network’s lifetime. Clustering can increase the lifetime of a network while also lowering energy use. Clustering will bring several similar sensors to one location for data collection and delivery to the Base Station (BS). The Cluster Head (CH) uses more energy when collecting and transferring data. The life of the WSNs can be extended, and efficient identification of CH can minimize energy consumption. Creating a routing algorithm that considers the key challenges of lowering energy usage and maximizing network lifetime is still challenging. This paper presents an energy-efficient clustering routing protocol based on a hybrid Mayfly-Aquila optimization (MFA-AOA) algorithm for solving these critical issues in WSNs. The Mayfly algorithm is employed to choose an optimal CH from a collection of nodes. The Aquila optimization algorithm identifies and selects the optimum route between CH and BS. The simulation results showed that the proposed methodology achieved better energy consumption by 10.22%, 11.26%, and 14.28%, and normalized energy by 9.56%, 11.78%, and 13.76% than the existing state-of-art approaches.

## 1. Introduction

WSNs comprise many low-energy sensors with substantial sink power responsible for establishing paths within specified transmission protocols [1]. WSNs are used in dynamic networks because of their ease of installation and quick synchronization with other sensors. WSN sensors can sense, gather, and send data in real-time [2]. Systematizing sensor networks into clustered frameworks has received much attention recently, resulting in many organizations developing their clustering methods [3].

Clustering is a basic methodology for designing energy-efficient, reliable, and flexible distributed sensor networks. Clustering decreases correspondence overhead, lowering energy consumption and impedance among the SNs [4]. The ultimate objective is to exploit the interaction between the sensors and eliminate the frequent repetition. Combining existing data with data acquired by sensors at CHs, the total amount of data transmitted to the sink may be drastically reduced [5].

While cluster-based sensor network connection and inclusion support protocols had previously been discussed, they have not been organized systematically [6]. Existing approaches for selecting CH nodes are based on various parameters, including the highest residual energy, area of the CH concerning alternate nodes, topological data, and last movement of the SN as a CH [7]. Most of these CH selection approaches ignore the network’s requirement for complete inclusion over long periods [8]. When removing nodes from regions that are sparsely populated with SNs, the passing of an SN issued in a densely populated territory has a lower impact on network inclusion. The importance of each SN to the incorporation endeavor may be quantified using an inclusion-conscious cost metric. This cost metric considers the node’s incredible energy and the inclusion outside its detecting range, measuring the node’s contribution to the network’s inclusion concerns [9].

In this sense, this article looked into the differences between energy-adjusted and inclusion-conscious sensor network association, with a particular focus on clustered WSNs. The traditional method is insufficient for effective CH selection. The main reason is that the node may shift from one cluster area to the other, making it difficult to choose a CH. As a result, the shortest path selection for RN is proposed to solve this problem, allowing data transfer even when the cluster zone changes [10]. Moreover, all these operations must be completed efficiently to avoid wasting the restricted sensor battery life. The sensor’s life cannot be increased by providing external or extra energy since most sensors are placed in difficult-to-reach locations. A network system with too many dead nodes may become paralyzed and unable to function correctly [11]. Therefore, it is not very easy for WSNs to develop energy-balanced and energy-efficient routing algorithms.

The lifespan of a network may be successfully extended by balancing the energy usage of nodes and enhancing energy efficiency. By separating nodes into many clusters, hierarchical clustering methods lengthen the network lifespan [12]. Clustering protocols attempt to identify the best CH set and rotate the function of the CH across all nodes for the optimization of node energy usage. The chosen CHs should significantly influence the clustering protocol’s performance.

Meta-heuristic optimization techniques are the best alternative for selecting appropriate CHs to extend the network’s lifespan. There are also many interesting applications of heuristics in different technical fields such as optimization in electric systems [13] and remote sensing models [14]. However, the algorithms face several common difficulties, such as fast convergence, local search concerns in the fitness function, and increased cost. Meta-heuristic algorithms are preferred when searching for the optimal solution becomes exhausting. Efficient meta-heuristic algorithms must include the solution space where there is a global optimum and develop novel and better solutions. There are meta-heuristic algorithms such as Particle Swarm Optimization (PSO) and Cuckoo Search that strive for global optimization (Exploration) and methods such as Simulated Annealing (SA) and Harmony Search Algorithm (HSA) that are restricted to local optima in the literature. For a better solution, a balance between exploration and exploitation is required. This prompted the combination of two popular meta-heuristic algorithms, MFA and AOA.

This research presents a hybrid Mayfly-Aquila optimization technique for an energy-efficient clustered routing protocol in WSNs to address earlier mentioned difficulties. To minimize energy consumption, this work proposes to include energy consumption as a node choosing the path. For that, the following tasks must be prioritized:

i. The shortest route between BS and CH because of its usage of less energy.
ii. The selection of CH between clusters.

MFA is a new evolving meta-heuristic that has a higher potential for finding the optimum solution than PSO and can identify more optimal solutions. Premature convergence may decrease the eventual solution’s quality in some cases. Combining an algorithm with high exploration skills with another approach with strong exploitation characteristics is possible. The MFA approach successfully explores the search space, whereas the AOA algorithm utilizes and improves existing feature subsets.

The following are the research’s key contributions:

- In the WSN, Mayfly is employed to determine the CH because of its excellent stability and low computing cost. Mayfly chooses the CH in this research based on many objective values, including residual energy, the distance between neighboring nodes, the distance towards the BS, density of the nodes, and node centrality.
- AOA determines the shortest route between CH and BS because of its capability of delivering speed detection of solutions. To address the constraint of the unpredictable convergence time, the AOA is optimized using residual energy, the density of the node, and distance.
- The network lifetime is increased due to the efficient CH selection and optimal path creation for data transmission. Furthermore, by reducing the energy usage of the nodes while transferring data packets, the total number of packets retrieved by the BS is enhanced.

The organization of this paper is as follows: Section 2 illustrates the existing works, Section 3 presents the proposed hybrid MFA-AOA algorithm, Section 4 discusses the result and discussion, and the conclusion and future work are presented in Section 5.

## 2. Related Works

In this section, the main related works in the area are analyzed to justify the need for the proposed work.

Murugadass, G. and Sivakumar, P. (2020) [15] presented a hybrid approach of Elephant Herding Optimization with a Cultural Algorithm for optimum CH selection (EHO-CA) to increase the lifespan. The advantages of the belief space provided by the cultural algorithm were used to define a separation operator that effectively created different local optimum solutions in the search space in this presented EHO-CA approach. Moreover, the addition of belief space assisted in providing the balance between an ideal exploitation and exploration process and better search abilities in the selection of optimal CH. The presented EHO-CA method improved the distinguishing qualities by combining separation and clan updating operators for optimal selection of CH to increase the network’s lifespan. The limitation of this proposed method was that it required more processing time.

For obtaining an extended network lifespan in sensor networks, Rambabu et al. (2019) [16] proposed a combined Artificial Bee Colony (ABC) and Bacterial Foraging (BF) (ABC-BF) based clustering method. The benefits of Bacterial Foraging Optimization were integrated with this proposed ABC-BFA approach for enhancing the local search ability of the ABC algorithm to achieve maximal exploitation and exploration of the parameters evaluated for the selection of CH. The proposed ABC-BFA approach was assessed in simulations utilizing a percentage of living and dead nodes and throughput using various SNs in the network. However, the packet drop in the proposed technique was high.

Tabatabaei et al. (2019) [17] investigated clustering SN to improve WSN lifespan. The Lion pride optimizer algorithm reduced energy consumption by grouping SN into clusters. For picking the best nodes as CHs, this method used two criteria: battery power range and distance from the sink. The remaining nodes that were not CHs are then joined to the CH closest to them. Clusters were produced in this manner. After clusters were constructed, data routing was facilitated by a direct virtual backbone, which was based in the sink node and made up of CHs. However, from the results, it was identified that the proposed method has a high processing overhead and attained less throughput.

Through the development of the Rider-Cat Swarm Optimization (RCSO) algorithm for beginning communication in SN, Shyjith et al. (2021) [18] built a platform for data transfer in WSN. The proposed RCSO method, which incorporated the ROA in the CSO algorithm, was used to build the CHs of the WSN nodes, and the CHs aid communication with the ideal CH, determined using the fitness function. The network was first established with initial energy, and the node mobility was regulated using the mobility model. During the setup phase, the CHs for data transmission from the nodes to the BS were determined using the clustering algorithm, which was created by determining the best threshold and CHs using the proposed RCSO method. Following the selection of the CHs, data transfer from the CH to the BS commenced. However, this method did not consider the Quality of Service (QoS) metric.

Poonguzhali and Ananthamoorthy (2020) [19] proposed a novel routing protocol based on Ant Colony Optimization (ACO) and HSA with optimum parameter selection. This research looked at how to send packets quicker without sacrificing data quality. The results demonstrated that the proposed strategy is more effective than other ways of increasing the network’s energy efficiency. However, the limitation of this work was that it only allowed for a meta-heuristic approach, which may have restricted utility in recovering prior network routing parameters.

Wang et al. (2020) [20] investigated the topic of reducing WSN energy consumption and proposed an energy-efficient routing protocol relying on an enhanced ABC algorithm. This research employed the modified ABC method to optimize the fuzzy C-means clustering and pick the optimal CH. In addition, this article employed a polling control access method based on busy/idle nodes, which conserved energy and improved network throughput. According to the simulation findings, the proposed method performed well in energy usage balance, power efficiency, network lifespan, network stability duration, and network throughput. Nevertheless, the developed algorithm could only be used in fixed networks.

Hassan et al. (2020) [21] proposed an enhanced energy-efficient clustering protocol to increase the lifetime of WSN-based IoT networks by addressing clustering structure issues that decrease protocol performance. By optimizing the clustering structure, the proposed technique lowered and balanced the power usage of nodes. As a result, the presented protocol was declared viable for networks with a longer lifespan requirement. In addition, the proposed method used a novel goal function to choose CHs in ideal locations. Though, uncertainty is a big concern with this model.

Al Mazaideh and Levendovszky (2021) [22] developed a method for determining the optimal values of direct detection of paradigm elements by multiple objective genetic algorithms. It was discovered that adjusting these factors will optimize energy efficiency while reducing the likelihood of reconstruction error. The proposed method achieved a fair balance between these two goals. They also devised a compressive sensing approach to conserve energy by decreasing the length of the sensing vector. When the proposed algorithms were evaluated to the performance of typical cluster selection algorithms, it was discovered that they had greater energy efficiency. However, the complexity of this technique was high.

Osamy et al. (2020) [23] introduced the hybrid Chicken Swarm- GA (CSGA) clustering algorithm, in which the CSO algorithm was updated to optimize the energy utilisation in WSNs, to enhance network lifespan. CSGA used a hierarchal order approach. The population was separated into three groups and then sorted according to fitness values to choose the finest nodes that function as CHs every round. To enhance population variety, CSGA used crossover and mutation mechanisms. In addition, the fitness function was created to reduce the overall amount of energy spent and the total number of times the chosen set of nodes worked as CHs. However, this method failed to address the network heterogeneity.

To tackle the optimal data transmission routing path in WSNs, Zhang et al. (2021) [24] proposed a unique and efficient Robust ACO (RACO) method based on ACO. The proposed method comprehensively considers power consumption, node distance, and connection security, improving ACO’s heuristic value. Furthermore, the numerical testing revealed that the RACO approach was resilient and improved network overhead performance without raising network design, operation, or communication. However, the proposed method has a higher computational cost.

To prolong the lifetime of heterogeneous WSNs, Li et al. (2020) [25] developed a Modified GA (MGA). In contrast to prior studies, MGA represented solutions using two-level organized chromosomes. The value of this chromosome was that it represented the exact scheduling of each set and the detailed energy allotment of each sensor. For the generation of the initial population, a greedy strategy was used. This approach has a temporal complexity. However, it considerably increases the pace of searching. Forward and backward mutation procedures were created as unique mutation operations. The forward mutation ensures chromosomal variety, whereas the backward mutation can aid in efficiently leaping out of local optima. However, scalability and mobility were not provided in this approach.

Li et al. (2019) [26] proposed a unique Load Balancing Ant-based routing protocol (LBAR) for WSNs. Under the limits of a limited energy supply, LBAR aimed to balance energy consumption, extend network lifetime, and speed up route-finding convergence. In the development of LBAR, a pseudo-random method was used to find routes, which speeds up the search for an efficient route and considers the energy balance. Furthermore, the pheromone trail updating mechanism took energy and path length information into account, resulting in a network lifespan extension. However, this method was unable to determine the cluster technique’s optimality.

Elsmany et al. (2019) [27] introduced the energy-efficient Scalable Routing Technique (SRA), a flexible, low-energy, and adaptive clustering hierarchical routing algorithm to sustain network lifespan despite network size growth. To minimize the stress on CH, SRA implemented a three-layer hierarchy structure and used multi-hop broadcast for intra-cluster communication. In this work, SRA was compared to different WSN routing schemes using network performance as a function of network size changes. According to simulation data, SRA outperformed benchmarking techniques through load balancing and energy efficiency for WSNs. However, this proposed method did not analyze the computational time and complexity.

Zhou et al. (2019) [28] presented Privacy-Preserving Data Aggregation (PPDA), a new aggregating technique that is both energy-efficient and secure. A sensor network was structured into an aggregation tree, and the leaf nodes of the tree were connected to form multiple chains, according to the proposed strategy. It reduced the number of leaf nodes in the basic aggregation tree. The simulation study findings revealed that the proposed algorithm is more efficient when compared to conventional aggregation techniques while maintaining superior privacy protection. PPDA performed well in terms of efficiency and accuracy of aggregation outputs. However, this technique was unable to develop a data aggregation model to reduce duplicate data.

It has been discovered that a great deal of effort has gone into controlling CH selection and improving data transfer across SN while the sink is moving. However, there has been a sprinkling of research that has concentrated on both aspects at the same time using any hybrid strategy. Using a hybrid method to combine the best features of both optimization techniques to obtain the best network performance.

## 3. Proposed MFA-AOA Algorithm

CHs are chosen using a formula based on the node’s various properties in traditional protocols. Even though these approaches are easy and simple to apply, they do not consider suitable criteria for choosing CHs. Although metaheuristic-based methods are more efficient in picking optimal CHs than classical techniques, they suffer from high time and computational difficulties since an iterative algorithm must be used to identify CHs at each round. The three categories mentioned above all have the disadvantage of not being application-specific. Therefore, the controllable parameters cannot be adaptively altered to meet the application’s needs. Though these procedures may provide acceptable results in certain situations, their effectiveness may be compromised in others. Furthermore, most present protocols do not use adequate criteria for selecting CHs (during the clustering phase) and forwarders (during the multi-hop routing phase). The proposed MFA-AOA is offered to address the problems mentioned earlier.

**Figure 1:**
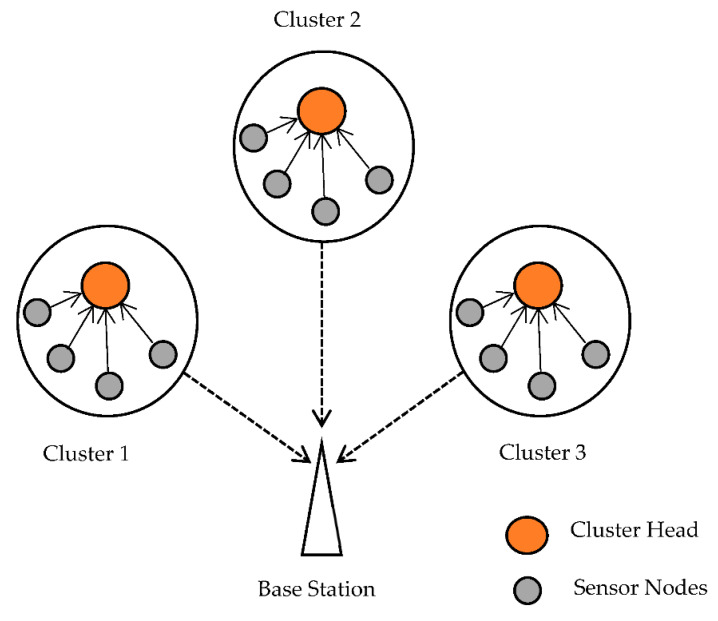
Construction of WSN.

By altering the components, an optimization strategy is utilized to reduce or maximize a function’s output. All viable options for this problem are referred to as possible solutions, and the best is referred to as the optimal solution. All swarm intelligence algorithms are population-based, which means that their iterative approach improves the position of individuals in the population and, as a result, their progress toward better positions. The network model, energy model, Mayfly optimization description, and Aquila optimization technique are all covered in this section. Figure 1 depicts the construction of the WSN. The following factors are used to develop the network model:

- All SNs starting energy and process time in a WSN are comparable.
- The Euclidean distance formula is used to compute the distance between the sensors
- The SNs are placed in the sensing region at random, and their position remains constant after installation
- The SNs send information to BS regarding the reserve energy and distance. An efficient CH selection method is used to choose CHs for all SNs based on that data. The routing procedure is then employed to find the optimal path between the CHs and the BS [29].

### 3.1. Energy Model

A 1st order radio model computes the transmitter and receiver energy. Equations (1) and (2) represent the amount of energy used to send and collect k bit packets over a given distance s [30].

(1) $$M_{TX}\left(k,s\right)=\{\begin{array}{c} k\times M_{elec}+k\times \epsilon_{ed}\times s^{2}\;if\;s\leq s_{0}\\ k\times M_{elec}+k\times \epsilon_{np}\times s^{4}\;if\;s>s_{0} \end{array}$$

(2) $$M_{RX}\left(k,s\right)=k\times M_{elec}$$

where $M_{elec}$ is the amount of energy released at the transmitter $\left(M_{TX}\right)$/receiver $\left(M_{RX}\right)$ and $s_{0}$ denotes the threshold distance. The following Equation (3) is used to compute the threshold distance.

(3) $$s_{0}=\sqrt{\frac{\epsilon_{ed}}{\epsilon_{np}}}$$

where and are the amplification energies for the free space $\epsilon_{ed}$ and multipath model $\epsilon_{np}$, respectively. The transmitter amplifier type determines these factors.

### 3.2. Mayfly Algorithm

A mathematical concept based on mayfly social behavior is named the mating process. Mayflies are considered adults in this method when they hatch from the egg, and only the fittest mayfly survives. The ideal solution to the issue is represented by the location of every mayfly in the search space. Each mayfly’s position in search space symbolizes a possible solution to the problem. Based on the fitness function, this algorithm identifies the optimal position, referred to as the best CH. This mathematical model considers nuptial dance and mayfly movement within a certain range. In addition, it determines the crossover value among mayflies to arrive at the ideal place.

Two sets of mayflies are first produced randomly and positioned in problem space as a potential solution signified by a dimensional vector. The velocity of a mayfly is defined as a variation in its location while evaluating fitness function g(x). Each mayfly’s flight path is in a dynamic direction. Each mayfly’s location is modified to the best ($P_{best}$) position attained by any mayfly in the swarm before ($g_{best}$). The best position is chosen as the basis for CH selection. The mayfly selects the best CH for all sensors based on node degree and centrality and the distance to its neighbors [31].

#### 3.2.1. Position of Male Mayflies

Male and female mayflies are initialized independently. Every mayfly modifies its position in response to its individual and its neighbors’ experiences. It is considered that the mayfly’s present location is $x_{j}^{t}$ and that the search space is designated as j at time t. When velocity $C_{jk}^{t+1}$ is added to the present location, position changes. It may be expressed as:

(4) $$x_{j}^{t+1}=x_{j}^{t}+C_{jk}^{t+1}$$

Here, velocity $C_{jk}^{t+1}=h*C_{jk}^{t}+y_{1}e^{-\alpha r^{2}}*p\left(Pbest_{jk}-C_{jk}^{t}\right)+y_{2}e^{-\alpha r^{2}}*g\left(gbest_{jk}-C_{jk}^{t}\right)$.

$y_{1}$ and $y_{2}$ are positive attraction constraints. $Pbest_{jk}$ is the optimal position of mayfly j had ever visited. Here, $r^{2}$ is the distance between male and female mayflies, h is the gravity coefficient and α is a fixed visibility coefficient. Pbest can be determined using minimization problems as follows:

(5) $$Pbest_{j}=\{\begin{array}{c} x_{j}^{t+1},if\;\left(x_{j}^{t+1}\right)<f\left(pbest_{j}\right)\\ iskeptsame,\;otherwise \end{array}$$

Similarly, at time step t, gbest can be given as:

(6) $$gbest\in \left\{pbest_{1},pbest_{1},\ldots \ldots ,pbest_{N}|f\left(pbest\right)\right\}\;=min\left\{f(pbest_{1}),f(pbest_{2}),\ldots \ldots ,f\left(pbest_{N}\right)\right\}$$

where, $P_{best1}$ is the first mayfly in the swarm and N is the total number of male mayflies.

#### 3.2.2. Movement of Female Mayflies

Male and female attraction is depending on the effectiveness of the existing solution. The best performing male attracts the best forming female, and so on until all partners are found. Moreover, the updated position $y_{j}^{t+1}$ for female mayflies can be expressed as follows:

(7) $$y_{j}^{t+1}=y_{j}^{t}+C_{jk}^{t+1}$$

where, $y_{j}^{t}$ is the present female mayfly position. The best female attracts the best male and the second best female to the second best male when considering female mayflies, velocity can be determined. The modified equation for a female’s velocity $C_{jk}^{t+1}$ is:

(8) $$C_{jk}^{t+1}=\{\begin{array}{c} if\;fitness\left(y_{j}\right)>fitness\left(x_{j}\right)\\ h*C_{XY}+y_{2}*e^{-\alpha r^{2}mf}*\left(Y_{jk}-X_{jk}\right)\\ \begin{array}{c} elseif\;fitness\left(Y_{jk}<X_{jk}\right)\\ h*C_{XY}^{t}+fl*r \end{array} \end{array}$$

where $C_{jk}^{t+1}$ is the velocity of jth element kth female mayflies at time t, $fitness\left(y_{j}\right)$ is the fitness value of female mayfly location, $fitness\left(x_{j}\right)$ is the fitness value of male mayfly location, the $Y_{jk}$ is the location of the female mayfly in dimension k at time t, $X_{jk}$ is the jth element of the location of the male mayfly in dimension k at time t, $y_{2}$ is the earlier determined attraction constant, h is the gravity coefficient, rmf is the Cartesian separation between the male and female mayflies, and r is a random number between [−1, 1]. In the scenario where a female is not attracted to a male $fl*r=fl_{0}\times \beta^{itr}$, then fl is a random walk coefficient. Here itr is the present iteration number and β is a random value Є [0, 1].

#### 3.2.3. Crossover

By initially recognizing a male mayfly and subsequently a female mayfly, a crossover operation happens. Best males breed with best females, and selection is based on fitness value. The equation below illustrates the offspring that result from a crossover.

(9) $$offspring1=r_{\mathrm{off}}*\left(1-r_{\mathrm{off}}\right)*female$$

(10) $$offspring2=r_{\mathrm{off}}*female+\left(1-r_{\mathrm{off}}\right)*male$$

This selection may be made at random or in accordance with the fitness function. Here, the male stands in for the male father and the female for the female parent. A value at random inside a certain range is called $r_{\mathrm{off}}$. Offspring velocities are initially set to zero.

### 3.3. Aquila Algorithm

One of the most studied birds in the world is the Aquila, which is famous for its hunting bravery. Male Aquila’s caught a lot more prey when they hunted alone. Aquila uses their speed and strong nails to hunt other animals. [32]. The Aquila optimization algorithm, which is a population-based technique, starts with a population of candidate solutions (Y), which is created randomly between the upper limit and lower bound of the given problem. The best-obtained solution is roughly selected as optimal in every iteration process.

In AOA, the optimization begins the improvement processes by producing a predetermined random population of potential solutions. The search criteria of the AO examine the reasonable locations of the best-obtained solution or the near-optimal solution through the repetition trajectory. The number of iterations needed by AO to reach the ideal solution is relatively low. It is clear from the solution’s trajectory that its magnitude and frequency are high in the initial iterations. They have almost totally vanished in recent versions. This demonstrates AOA’s strong ability for exploration in the early iterations and its strong ability for exploitation in the last iterations. This behavior indicates that there is a good likelihood that AOA will find the optimal solution.

Four different procedures are used to catch the prey in the Aquila algorithm [33].

#### 3.3.1. Approach 1: Extended Exploration

Initially, the Aquila recognizes the prey’s position and flies around looking for it.

(11) $$Y_{i}\left(t+1\right)=Y_{best\;}\left(t\right)\times \left(1-\frac{t}{T}\right)+Y_{M\;}\left(t\right)-Y_{best\;}\left(t\right)*rand$$

where $Y_{i}\left(t+1\right)$ denotes the ith individual’s location at iteration t+1. The best location at the current iteration is represented by $Y_{best\;}\left(t\right)$. The mean locations of all individuals at the current iteration are represented by $Y_{M\;}\left(t\right)$.

(12) $$Y_{M}\left(t\right)=\frac{1}{L}\sum_{i=1}^{L}Y_{i}\left(t\right)$$

where $Y_{i}\left(t\right)$ denotes the location of the ith individual at iteration t, and L is the swarm population size. T denotes the maximum permitted iteration number, while rand is the random number in a Gaussian distribution between 0 and 1. Based on this condition, if $t\leq \left(\frac{2}{3}\right)T$, the exploration phases will be activated; else, the exploitation phases will be carried out, and the AOA algorithm can switch from exploration phases to exploitation phases utilising different behaviours.

#### 3.3.2. Approach 2: Narrowed Exploration

The next approach is narrowed exploration. Continuing with the exploration technique, the Aquila would fly surrounding the prey and ready the land once it located it.

(13) $$Y_{i}\left(t+1\right)=Y_{best\;}\left(t\right)\times Levy\;\left(E\right)+Y_{R\;}\left(t\right)+\left(x-y\right)*rand$$

where E denotes the dimensionality of the problems to be solved. Levy (E) stands for Levy flights, which are computed as follows:

(14) $$Levy\;\left(E\right)=p\times \frac{\alpha \times \beta }{{\left|\delta \right|}^{\frac{1}{\gamma }}}$$

where α, δ are random values between 0 and 1, and *p* = 0.01 is a constant parameter. β is determined in the following way:

(15) $$\beta =\frac{\Gamma \left(\left(1+\gamma \right)\times sin\left(\frac{\pi \gamma }{2}\right)\right)}{\Gamma \left(\left(\frac{1+\gamma }{2}\right)\times \gamma \times 2^{\frac{\gamma -1}{2}}\right)}$$

where γ is a fixed value of 1.5. Γ indicates the gamma function. At the current iteration, $Y_{R\;}\left(t\right)$ represents a randomly picked candidate. The spiral form is represented by *x* and *y*, which are computed as follows:

(16) x=rcosθ

(17) y=rsinθ

(18) $$r=r_{1}+V\times E_{1}$$

(19) $$\theta =-\omega \times E_{1}+\theta_{1}$$

(20) $$\theta_{1}=\frac{5\pi }{2}$$

where $r_{1}$ is a fixed value ranging from 1 to 20 and V is a small value fixed to 0.00464. $E_{1}$ is an array of integer numbers ranging from 1 to the whole length of the issues. The number ω = 0.004 is a constant.

#### 3.3.3. Approach 3: Extended Exploitation

When the Aquila fails to detect the target during the exploitation operation, they may re-initialize themselves; they then update their locations using the equation below.

(21) $$Y_{i}\left(t+1\right)=\sigma \times \left[Y_{best\;}\left(t\right)-Y_{R\;}\left(t\right)\right]+\epsilon \times \left[\left(UB-LB\right)\times rand+LB\right]\;$$

The definitional domain of the presented issue is [LB,UB]. σ and ϵ are two little fixed integers.

#### 3.3.4. Approach 4: Narrowed Exploitation

When the Aquila comes near the prey, they use the following equation to restrict their exploitation.

(22) $$Y_{i}\left(t+1\right)=Q\times Y_{best\;}\left(t\right)-H_{1\;}\times Y_{1\;}\left(t\right)\times rand-H_{2\;}\times Levy\;\left(E\right)+rand\times H_{1\;}\;$$

The AO’s different tracking motions during the session are represented by $H_{1}$, which is computed using Equation (24). The flight slope of the AO utilized to follow the prey during the escape from the starting position to the final position (t), which is derived using Equation (25), is represented by $H_{2\;}$ by decreasing values from 2 to 0. Q is a quality function that is employed to optimize the search plan, and it is derived using the equation below.

(23) $$Q=t^{\frac{2\times rand-1}{{\left(1-T\right)}^{2}}}$$

(24) $$H_{1\;}=2\times rand-1$$

(25) $$H_{2}=2\times \left(1-\frac{t}{T}\right)$$

### 3.4. Proposed Method

**Figure 2:**
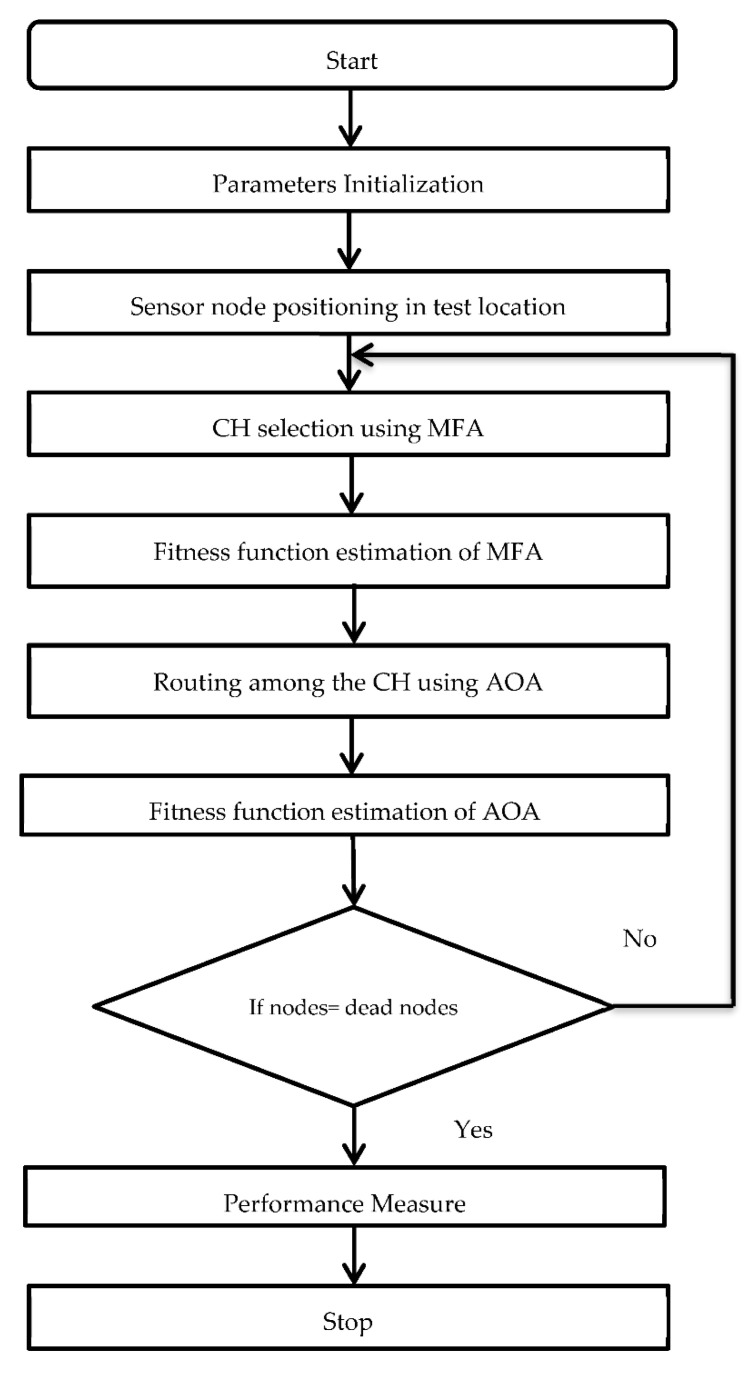
Flowchart of the proposed MFA-AOA.

The proposed method works based on the characteristics of two algorithms: one for selecting the CH and the other for network routing. First, the Mayfly algorithm identifies appropriate sensors and Aquila determines the best path between the CH and the BS. Following that, the CHs send the data obtained to the BS through the path generated by the Aquila algorithm. Figure 2 illustrates the overall procedure of the proposed technique. The proposed MFA-AOA optimization approach has a powerful hybrid algorithmic framework based on the behavior of the mayfly and Aquila. The best results are attained when this technique is paired with a routing protocol.

Cluster maintenance is one of the most critical stages in this analysis for balancing the load amongst clusters. Due to inter-cluster communication, clusters closer to the BS consume too much energy. Therefore, the cluster maintenance stage is necessary to prevent node failure. It increases the lifespan of data transmission from the SN to the BS. The MFA algorithm is re-initialized to cluster the network if the CH’s residual energy exceeds the threshold level. The CHs are then chosen using the clustering technique, and the AOA is utilized to find the routing path between the CHs and the BS. The MFA algorithm is used in this proposed technique to conduct an effective CH selection. The CHs are chosen based on five criteria: node level, node density, distance from neighbors, distance from the BS, and residual energy. These characteristics are used to choose the best CH from the available nodes.

#### 3.4.1. Evaluation of MFA Fitness Function

The best CH is chosen from the network’s collection of sensors using the identical fitness function of MFA. To avoid using a dead node as a CH during the clustering process, the residual energy taken into account by the fitness function is used. The next step is to choose the best CH to reduce the nodes’ energy consumption using the distance between the nodes and the distance from the potential CH to the BS. The node degree is taken into account when choosing the CH with the lowest normal nodes in order to keep the node for the next rounds. Additionally, the cluster members’ transmission distances to CH are reduced due to the enhanced centrality of the cluster member. The fitness factors have been optimised for the best possible outcomes. The developed model fitness function is for minimising the energy usage and thereby extends network lifespan. The fitness function initialization parameters taken into account for CH selections are explained in the following sections.

#### 3.4.2. Residual Energy

CH chooses its primary factor depending on the remaining energy in each round. The CH rotation is based on the nodes’ residual energy, which results in energy distribution across the network. Maximum energy is used to pick CH in this proposed network. This metric represents the ratio of residual energy to the total energy. It can be expressed as follows:

(26) $$g_{1}=\frac{1}{\sum_{i=1}^{R}\frac{W_{r}}{W_{T}}}$$

where R stands for the maximum number of nodes. $W_{r}$ stands for residual energy, while $W_{T}$ stands for total energy spent. Decreased value of $g_{1}$ possibility of picking that node as CH gets minimal.

#### 3.4.3. Distance

The distance between nodes and BS determines the amount of energy consumed. Although the separation across the node and the BS are shorter, each node consumes more energy. Conversely, when the distance between the node and the BS is small, every node’s energy consumption is small. As a result, routing protocols use the distance factor as a primary measuring metric. The following is an example of a distance factor:

(27) $$g_{2}=\sum_{i=1}^{R}\frac{S_{\left(R\left(i\right)-B\right)}}{S_{avg\left(R\left(i\right)-B\right)}}$$

The distance between the ith node and BS are represented by $S_{\left(R\left(i\right)-B\right)}$.The average distance between ith node and BS are represented by $S_{avg\left(R\left(i\right)-B\right)}$. For CH selection, this fitness measure should be at its maximum.

#### 3.4.4. Rate of Energy Consumption

$S_{cr}$ or the energy consumption rate is a key component in determining whether a node is suitable for CH selection. It is the difference between the node’s starting energy and the residual energy of the node after the first round. The variation between initial and residual energy is considered in the computation. As a result, $S_{cr}$ is measured and compared to the voltage threshold. If the computed value is lesser than the threshold value, that node is designated as CH. Otherwise, the node remains a member node. The $S_{cr}$ is computed as follows:

(28) $$g_{3}=\sum_{i=1}^{R}\frac{\left(S_{(c\left(i\right)}-S_{(sc\left(i\right)}\right)}{S_{\left(c\left(i\right)\right)}}$$

$S_{\left(c\left(i\right)\right)}$ denotes the present location energy value of the ith node in the preceding round and indicates the energy used by the ith node in the current round. When the number of iterations increases by one, the energy from the last round becomes the new round’s beginning energy. The node with the minimum energy within the cluster is not considered while computing the average threshold value of ECR. A node that uses a lot of energy is not suitable for CH selection.

#### 3.4.5. Node Degree

It describes the number of SNs associated with each CH. The CHs with higher clusters lose their energy over longer periods of time, hence the CHs with fewer sensors are chosen. Equation (29) expresses the node degree.

(29) $$g_{4}=\sum_{i=1}^{R}J_{i}$$

where $J_{i}$ denotes the number of SNs.

#### 3.4.6. Node Centrality

Node centrality describes how far a node is from its neighbours.

(30) $$g_{5}=\sum_{i=1}^{R}\frac{\sqrt{\left(\sum_{j\in m}s^{2}\left(i,j\right)/n\left(i\right)\right)}}{Network\;Dmensions}$$

where n(i) denotes the number of neighboring SNs.

Each objective value is given a weight value. In this instance, a single objective function replaces the multiple objectives. $\alpha_{1},\alpha_{2},\alpha_{3},\alpha_{4}\;\mathrm{and}\;\alpha_{5}$ are the weighted values. The Equation (32) depicts the single objective function.

The fitness function equation of MFA is as follows:

(31) g(x)=Max{G}

(32) $$G=\frac{1}{\alpha_{1}g_{1}+\alpha_{2}g_{2}+\alpha_{3}g_{3}+\alpha_{4}g_{4}+\alpha_{5}g_{5}}$$

where, $\sum_{i=1}^{5}\alpha_{i}=1,\;\alpha_{i}\in \left(0,1\right)$.

The values of $\alpha_{1},\alpha_{2},\alpha_{3},\alpha_{4}\;\mathrm{and}\;\alpha_{5}$ are 0.33, 0.25, 0.20, 0.12 and 0.1 respectively. To prevent the node failure as a CH, the $\alpha_{1}$ is given consideration for taking residual energy as a higher priority. Then, the $\alpha_{2}$ and $\alpha_{3}$ are given second and third priority concerns to locate the CH from the BS with the shortest distance possible, hence minimising energy loss. The node degree is specified as the fourth priority to choose the CH with the lowest node degree $\alpha_{4}$. In order to strengthen the connection between the CH and cluster members, the node centrality $\alpha_{5}$ is also given final priority.

To determine the optimal solution, the fitness values of the updated and initial mayflies are compared. After analyzing each fitness value, the best mayfly in the present location is selected as CH. The optimum fireflies’ fitness value is also compared to the $g_{best}$ to achieve the optimal solution.

### 3.5. Routing Using the Aquila Algorithm

Data transmission to the BS in a clustered WSN is energy-efficient due to suitable routing between CHs. After figuring out the best paths for every CH in the network, this study uses AOA, as explained in Section 3.3, to identify the best routes for sending raw data to BS.

#### 3.5.1. Initialization

Each Aquila in AOA-based routing reflects a path from each CH that the MFA has chosen to the BS, which is defined as the prey area. Each Aquila has a dimension equal to the number of CHs chosen by MFA. Every Aquila position in the network is initialised in the solution vector to represent a subsequent SN towards the BS. Assuming $A_{i}$ represents the ith Aquila in the network, each Aquila’s position is assigned at random SNs from CHs.

#### 3.5.2. Fitness Function for Routing

The fitness function uses three factors to determine the best path from *CH* to *BS*. The first constraint determines the distance between *CH* and *SN*, the second determines the distance between *SNs* and *BS*, and the third determines the residual energy of *SNs*. Below are the derivations of these three parameters.

(a) Distance between *CH* and Sensor nodes:

$P_{sCHSN}$ stands for a function that calculates the distance between the *CH* and its chosen SNs at random. Equation (33) contains the derivation of this function.

(33) $$P_{sCHSN}=\sum_{i=1}^{x}dist(CH_{i},SN\left(CH_{i}\right)$$

where $SN\left(CH_{i}\right)$ represents sensor node of ith *CH*.

(b) Distance between *SNs* and *BS*:

A function called $P_{sSNBS}$ is used to calculate the separation between the *SNs* and *BS* that were chosen at random. It is energy-efficient to send data from *CH* to *BS* over *SNs* if the distance between those two devices is kept to a minimum. This function’s derivation is provided in Equation (34).

(34) $$P_{sSNBS}=\sum_{i=1}^{x}dist(SN\left(CH_{i}\right),BS)$$

(c) Residual energy of *SNs*:

The function $RE_{SN}$ is used to determine the residual energy of the subsequent *SNs*. If *SNs* have enough remaining energy, it would be advantageous to be selected as *SN* by a *CH*. Using Equation (35), the residual energy of *SNs* is computed as:

(35) $$RE_{SN}=\sum_{i=1}^{x}RE_{SN}\left(CH_{i}\right)$$

As the overall fitness function $AO_{routing}$ is a minimization problem, the calculation of $P_{RESN}$, which reflects a minimization of $RE_{SN}$, is done using Equation (36).

(36) $$P_{RESN}=\frac{1}{RE_{SN}}$$

The concluding fitness function combining the parameter functions described in Equations (33)–(35) is regarded as a minimization problem and is determined using Equation (37), which is also a minimization problem.

(37) $$\mathrm{Minimize}\;AO_{routing}=\frac{1}{2}\left[P_{sCHSN}+P_{sSNBS}+P_{RESN}\right]$$

#### 3.5.3. Updating AOA

As stated in Section 3.3, AOA employs four processes to identify the optimal solution. Equation (11) is utilised in the expanded exploration phase to update the locations of the AOA, and Equation (37) is employed to compute fitness. The process is carried out until the iteration stopping requirement is not met if the updated population’s fitness is higher than the previous one. Equations (13), (21) and (22) are also used to update the locations in the other three stages of narrowed exploration, extended exploitation, and narrowed exploitation. The top positions are determined using the best fitness value for each phase. The best optimal path to the BS is discovered once all AOA iterations have been completed. This energy-efficient channel enables sending aggregated data to the BS.

Algorithm 1 shows the pseudo-code for the proposed hybrid clustering algorithm in MFA-AOA. Initially, a random population $Q_{0}$ is created and split into two groups: $Q_{1}$ (MFA) and $Q_{2}$ (AOA). The whole populations of MFA and AOA ($Q_{1}$ and $Q_{2}$) are assessed by the suggested multi-objective function during each iteration of MFA-AOA. The two populations $Q_{1}$ and $Q_{2}$ are then shuffled and reassembled into new $Q_{1}$ and $Q_{2}$ populations, which are then chosen at random. The procedure is continued until it reaches the maximum number of iterations. When the proposed MFA-AOA is finished, the algorithm’s global best solution is decoded to find the relevant CHs.
**Algorithm 1:** Proposed hybrid MFA-AOA Algorithm#**MFA optimization****initialize** population of male mayflies **initialize** population of female mayfliesCalculate the fitness and update $Q_{1best}$Find $p_{best}$$\;\mathrm{and}\;g_{best}$while (iteration<max_iteration)  Update male mayfly’s velocity using Equation (4)  Update female mayfly’s velocity using Equation (8)  Perform crossover using Equation (9)  Distinct male and female mayfly  Replace the worst solution with the latest one  Compute the fitness function of the best mayfly using Equation (32)  Update $p_{best}$$\;\mathrm{and}\;g_{best}$**End while****Return**$\;Q_{1best}$#**AOA optimization****initialize** population of $Q_{2}$:**While**$\left(t\leq \left(\frac{2}{3}\right)max_{2}\right)do$  Calculate fitness and update $Q_{2best}$**For** (i = 1:M) **IF**
rand<0.4  Update $Q_{2newi}$ by using Equation (11) #Extended Exploration**Else**;  Update $Q_{2newi}$ by using Equation (13) #Narrowed Exploration**End If**  Compute the fitness value of $Q_{2newi}\;$$\mathrm{and}\;Q_{2i}$ using Equation (37)   Update $Q_{2i}$   **End For****Else:****For** (i = 1:M)  **IF** rand<0.4  Update $Q_{2newi}$ by using Equation (21) # Extended Exploitation**Else**;  Update $Q_{2newi}$ by using Equation (22) # Narrowed Exploitation**End If**  Compute the fitness value of $Q_{2newi}\;$$\mathrm{and}\;Q_{2i}$ using Equation (37)   $\mathrm{Update}\;Q_{2i}$  **End For****Else if**    t=t+1**End while****Return**$\;Q_{2best}$Post-process results and discussions

BS constantly monitors the remaining energy of the nodes to prevent node failure during data transmission. The AOA method finds the best transmission path from the SN to the BS through CH. It finds the shortest way to lower the nodes’ energy consumption. This MFA and AOA-based optimum CH selection and route development created an energy-efficient WSN. As a result, an energy-efficient WSN is utilized to increase the total number of packets transferred to the BS at data transmission, extending the network lifetime. Algorithm 1 shows the pseudo-code for the proposed hybrid MFA-AOA algorithm.

## 4. Results and Discussion

The proposed MFA-AOA optimized protocol, as well as the existing Hybrid Red Deer-Simulated Annealing (RDSA) [34], Genetic Algorithm-PSO (GAPSO) [35], Clan Separator Elephant Herding Optimization operator (CSEHO) [36], and Moth Flame-GA (MFGA) [37] techniques, are tested using MATLAB 2019b. The simulation setup consists of 500 SNs distributed randomly in a network area of 1500 × 1500 square meters, with one sink node at 500 m. Three thousand five hundred rounds were employed in the implementation process. In addition, Table 1 shows the simulation setup used to implement the proposed MFA-AOA optimized protocol.

The proposed MFA-AOA optimized protocol and the existing RDSA [34], GAPSO [35], CSEHO [36], and MFOGA [37] techniques are first examined in terms of network lifetime, energy usage, throughput, and packet delay with SN density.

**Figure 3:**
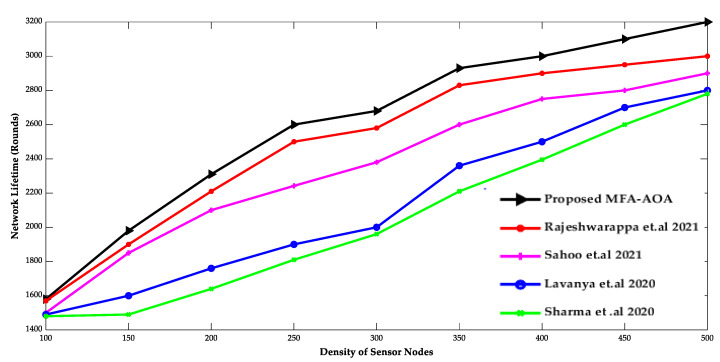
Density of sensor nodes vs. Network Lifetime [34,35,36,37].

**Figure 4:**
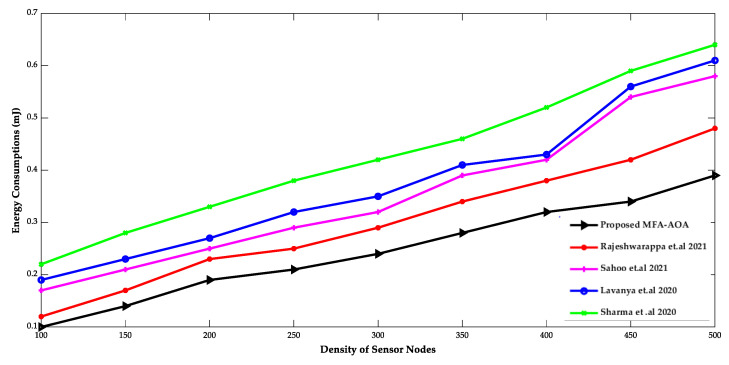
Density of sensor nodes vs. Energy consumption [34,35,36,37].

Figure 3 and Figure 4 show the comparison of the proposed MFA-AOA optimized routing protocol with existing protocols in terms of network lifespan, energy usage, and density of SN. Since it included the advantages of MFA into the AOA for determining the superior nodes of the network as CH, the network lifespan of the proposed MFA-AOA optimized protocol and the existing methods demonstrate a better life with a systematic increment of SNs. Furthermore, this dominance of the proposed MFA-AOA optimized protocol is achieved by replacing MFA’s searching phase with AOA’s hunting phase throughout the search space to preserve the trade-off between exploitation and exploration.

The proposed MFA-AOA optimized protocol and the existing schemes’ energy consumptions are shown to grow consistently as SN density increases. However, because it comprises different techniques that combine candidate solutions into prospective offspring solutions, the proposed MFA-AOA optimized protocol can sustain energy consumption. This analysis of future offspring focused on identifying and eliminating the worst solutions that avoid the least critical SNs from being chosen as CHs. The proposed MFA-AOA optimized protocol enhances network lifespan by 9.78%, 11.24%, and 14.25% with varied density of SNs, compared to the RDSA [34], GAPSO [35], CSEHO [36], and MFOGA [37] techniques. With different densities of SNs, the proposed MFA-AOA optimized routing protocol reduces energy consumption by 10.22%, 11.26%, and 14.28%, which is better than the existing systems.

**Figure 5:**
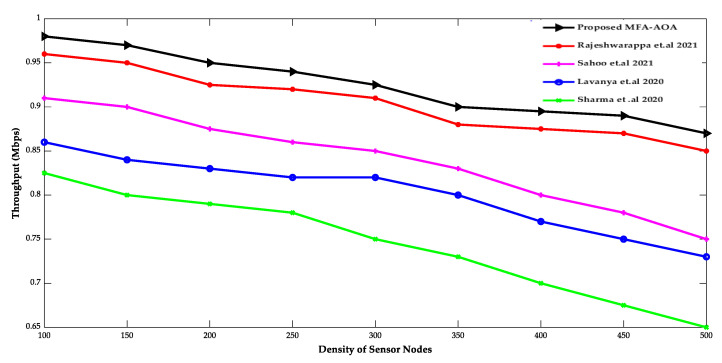
Density of sensor nodes vs. throughput [34,35,36,37].

**Figure 6:**
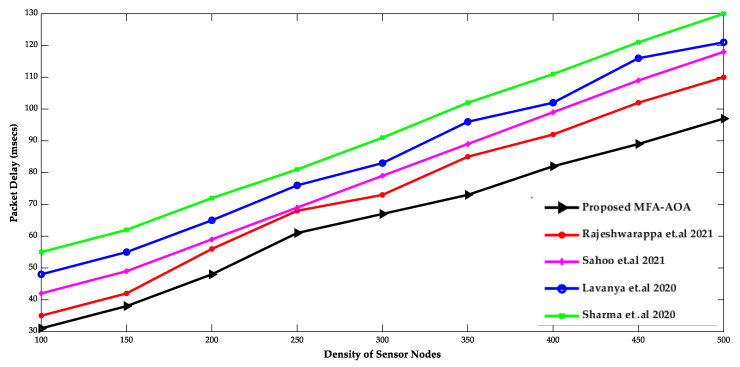
Density of sensor nodes vs. Packet Delay [34,35,36,37].

Figure 5 and Figure 6 compare proposed MFA-AOA optimized protocols with existing protocols in terms of throughput and packet delaying of SN densities. The proposed MFA-AOA optimized protocol’s throughput is verified to be superior to the RDSA [34], GAPSO [35], CSEHO [36], and MFOGA [37] techniques with scaling increases in the density of SNs. Furthermore, this capacity of the proposed protocol is improved proportionally because of the benefits of hunting, which assisted in better CH selection and prevented packet drop to an expected level.

Similarly, the proposed MFA-AOA optimized protocol achieves significantly lower packet latency. The selection of prospective CH in the network was always ensured by categorizing solutions and creating important offspring solutions. As a result, the proposed MFA-AOA optimized protocol enhances throughput by 11.36%, 12.79%, and 14.45%, respectively, when compared to the RDSA [34], GAPSO [35], CSEHO [36], and MFOGA [37] techniques. Furthermore, with variable densities of SNs, the proposed MFA-AOA optimized protocol reduces packet latency by 9.27%, 10.34%, and 12.78%, which is better than the existing systems. The proposed MFA-AOA optimized protocol, as well as the RDSA [34], GAPSO [35], CSEHO [36], and MFOGA [37] techniques, are compared in terms of the number of living and dead SNs, throughput, and mean residual energy over several rounds.

**Figure 7:**
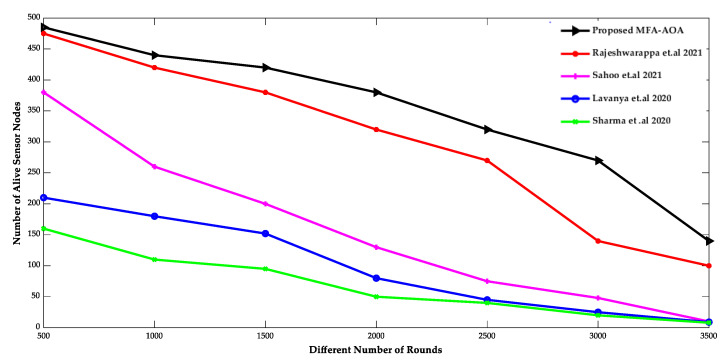
Different Number of Rounds vs. Alive sensor nodes [34,35,36,37].

**Figure 8:**
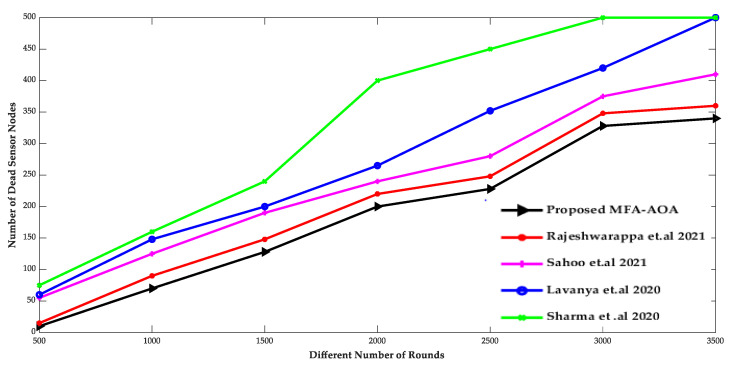
Different Number of Rounds vs. Dead sensor nodes [34,35,36,37].

The comparison of the number of living SNs and the number of dead SNs deployed in the network of the proposed MFA-AOA optimized protocol and the RDSA [34], GAPSO [35], CSEHO [36], and MFOGA [37] techniques are shown in Figure 7 and Figure 8. As the network’s energy is saved by choosing only the possible SNs as CH throughout the clustering process, the proposed MOA-AOA optimized routing protocol is superior to the state-of-art schemes in terms of the number of alive nodes maintained. This selection technique eliminates unnecessary clustering because the worse SNs are not picked as CH nodes. It keeps the network’s energy at a reasonable level by preventing wasteful energy-draining in the SN. As a result, the proposed MFA-AOA protocol maintains the number of living nodes by 12.54%, 13.63%, and 15.28%, respectively, compared to the RDSA [34], GAPSO [35], CSEHO [36], and MFOGA [37] techniques. Furthermore, the proposed MFA-AOA protocol reduces the number of dead nodes by 11.46%, 13.28%, and 14.63% with varied rounds, which is superior to the existing methods.

**Figure 9:**
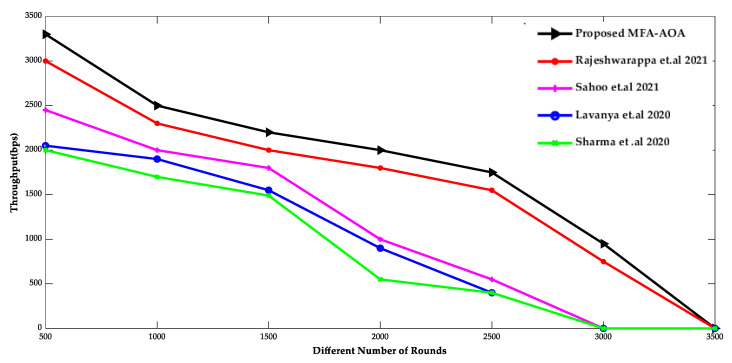
Different Number of Rounds vs. Throughput [34,35,36,37].

**Figure 10:**
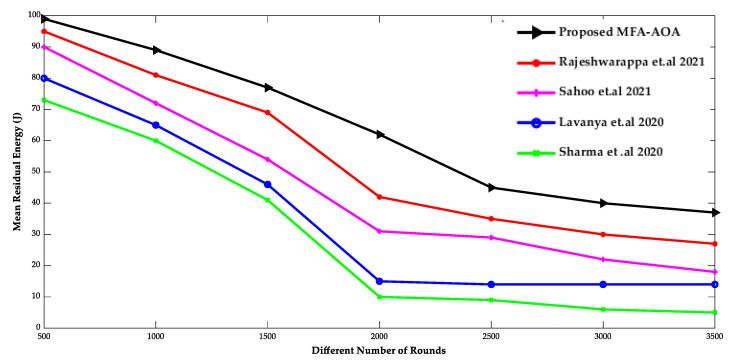
Different Number of Rounds vs. Mean Residual Energy [34,35,36,37].

Figure 9 and Figure 10 show the comparison of throughput and mean residual energy of the proposed MFA-AOA optimized protocol as well as the RDSA [34], GAPSO [35], CSEHO [36], and MFOGA [37] techniques with different rounds.

The proposed MFA-AOA strategy outperforms the other schemes in terms of throughput because the local search ability initiated by MFA into AOA allows for enhanced CH selection. MFA’s local search capability is also essential to minimize local points of optimization problem and stagnation to the maximum level, regardless of the number of implementation cycles performed. As a result, the proposed MFA-AOA protocol enhances throughput by 8.52%, 10.98%, and 12.69%, respectively, compared to the existing techniques. In addition, the mean residual energy of the proposed MFA-AOA protocol improves by 9.56%, 11.78%, and 13.76% with a varied number of rounds, which is superior to the benchmarked methods.

**Figure 11:**
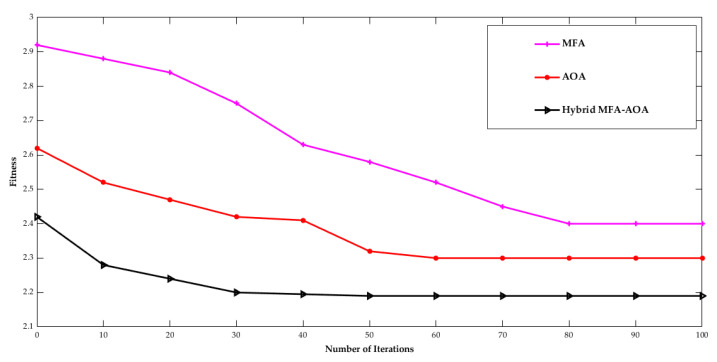
Number of iterations vs. Fitness.

Figure 11 shows the convergence rate of the proposed MFA-AOA algorithm for 100 nodes. The performance of MFA, AOA, and hybrid MFA-AOA is calculated at the 15th iteration. Hybrid MFA-AOA provides an optimal path with the minimum time consumption compared to the individual MFA and AOA algorithm performances.

Moreover, the number of rounds until the first node death, half of the node death, and the last node death in the network are explored, and the proposed MFA-AOA protocol is compared with the existing state-of-art methods [34,35,36,37].

**Figure 12:**
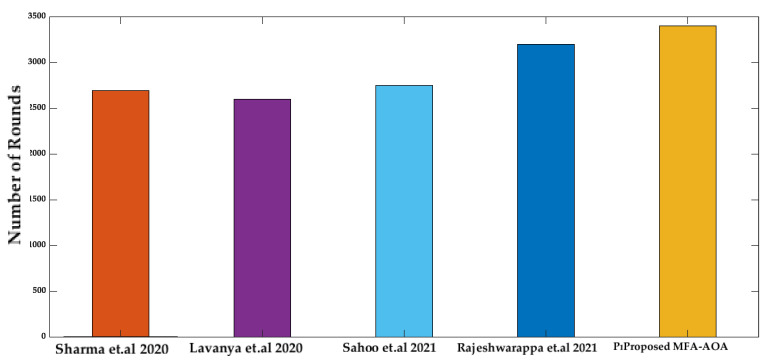
Number of rounds until first node death [34,35,36,37].

From Figure 12, the proposed MFA-AOA protocol experiences the first node death at round 3400, whereas the first SN death of the compared techniques occurs between rounds 2695 and 3200. The adaptive technique included by MFA into the basic AOA scheme throughout the exploitation process is primarily responsible for the proposed MFA-AOA protocol’s remarkable performance.

**Figure 13:**
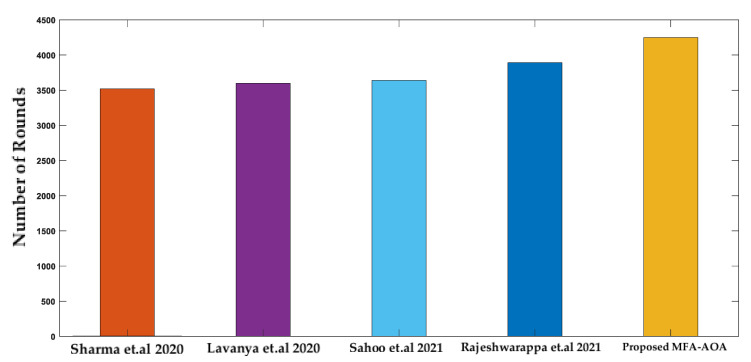
Number of rounds until half nodes death [34,35,36,37].

**Figure 14:**
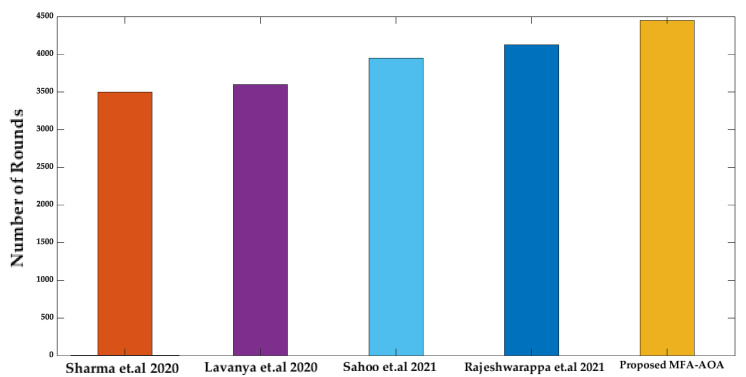
Number of rounds until last node death [34,35,36,37].

Figure 13 shows that the proposed MFA-AOA protocol is estimated to be half of the node’s death by round 4250, whereas the other techniques have half of the node’s death between 3520 and 3892 rounds. The proposed AOA protocol’s critical performance is achieved by including a flexible exploitation mechanism that varies based on the potentiality of solutions found during the mating phase. Furthermore, Figure 14 shows that the proposed AOA protocol’s last node death occurs at 4450 rounds, whereas the compared schemes occur between 3500 and 4126 rounds.

This extraordinary capacity of the proposed AOA protocol can be seen owing to the policy implemented for keeping the degree of exploration and exploitation at a predictable level irrespective of the number of rounds in implementation. The suggested AOA’s significant performance is mostly due to the numerous selections of random sample solutions generated from the same cluster in which the commander solution exists and from the remaining clusters in the network. Due to the great searching efficiency of MFA combined with the dynamic exploratory character of AOA, it can be stated that the proposed hybrid MFA-AOA algorithm outperforms other existing algorithms.

In comparison to the standardised schemes employed for the investigation, Table 2 shows the best, worst, mean, median, and standard deviation in the time complexity of the proposed MFA-AOA protocol. The findings clearly demonstrated that the presented MFA-AOA, when measured in terms of best, worst, median, and SD in time complexity, is superior since it included the benefits of MFA and AOA in the optimal CH and path selection, hence reducing the difficulty incurred in CH selection.

## 5. Conclusions

For ensuring lifespan enhancement and energy stability in WSNs, the MFA-AOA optimized method was presented in this paper: a combination of the global optimization capabilities of the MFA optimization method with the local optimization ability of AOA. This inclusion of AOA in MFA aids in achieving a balance between exploration and exploitation in the selection of CH for long-term energy stability. Meta-heuristic qualities of MFA and AOA are inherited in this clustering algorithm for locating essential CHs and the best BS placement to improve energy efficiency. In comparison to the existing RDSA, GAPSO, CSEHO, and MFOGA methods, the simulation results of the proposed MFA-AOA-based routing protocol enhanced network lifespan by 9.78%, 11.24%, and 14.25%.

Furthermore, with different densities of SNs, the proposed MFA-AOA routing protocol reduces energy consumption by 10.22%, 11.26%, and 14.28%, which is better than the existing methods. Furthermore, it increased the median number of living nodes by 12.54%, 13.63%, and 15.28%, respectively, while maintaining median normalized energy of 9.56%, 11.78%, and 13.76%, which was superior to the existing schemes. Therefore, it has been determined that the proposed MFA-AOA should be used to endorse practical issues as part of the future scope of work. Furthermore, optimization techniques based on self-adaptive approaches may be employed to address the network’s energy consumption problem.

## Figures and Tables

**Table 1:** Simulation parameters of the proposed MFA-AOA protocol.

Simulation Parameters	Values
Network Area	1500×1500 square meters
Number of Sensor Node	500
Number of Nodes used for implementation	3000
Location of the sink node	(500 m, 500 m)
Size of the packets	10,000 bits
Initial energy of the node	2 Joules
Range of nodes	20–30 meters
$E_{Amp}$	100 pJ/bit/square meters
$E_{Elec}$	5 nJ/bit
EDA	50 nJ/bit/signal
Maximum Network Throughput	1 Mbps data

**Table 2:** Time complexity comparison of proposed MFA-AOA with existing schemes.

	Best	Worst	Mean	Median	SD
**Proposed MFA-AOA**	1.6842	2.9245	1.8947	1.9084	0.1610
**RDSA** [34]	1.9873	3.4122	2.1115	2.1015	0.1911
**GAPSO** [35]	2.2741	5.6486	2.2340	2.2115	0.2042
**CSEHO** [36]	2.3941	12.1641	2.6163	2.6141	0.2141
**MFOGA** [37]	2.5123	13.2462	3.1211	3.024	0.3042

## Data Availability

Not applicable.

## Abbreviations

**Symbols**	**Meanings**
$M_{TX}$	Transmitter
$M_{RX}$	Receiver
k	Number of bits
s	Distance
$M_{elec}$	Amount of energy
$s_{0}$	Threshold distance
$\epsilon_{ed}$	Energies for the free space
$\epsilon_{np}$	Multipath model
g(x)	Fitness function
$P_{best}$	Best position
$P_{best1}$	First mayfly in the swarm
N	total number of male mayflies
$x_{j}^{t}$	Male mayfly’s present location
j	Search space
t	Time
$C_{jk}^{t+1}$	Velocity
$x_{j}^{t+1}$	Updated position of male mayfly
$y_{1}\;\mathrm{and}\;y_{2}$	Positive attraction constraints
$Pbest_{jk}$	Optimal position of mayfly had ever visited
$r^{2}$	Distance between male and female mayflies
h	Gravity coefficient
α	Fixed visibility coefficient
$y_{j}^{t}$	Female mayfly’s present location
$y_{j}^{t+1}$	Updated position of female mayfly
$fitness\left(y_{j}\right)$	Fitness value of female mayfly location
$fitness\left(x_{j}\right)$	Fitness value of male mayfly location
$Y_{jk}$	The location of the female mayfly in dimension k
$X_{jk}$	jth element of the location of the male mayfly in dimension k
rmf	Cartesian separation between the male and female mayflies
r	Random number between [−1, 1]
fl	Random walk coefficient
itr	present iteration number
β	Random value Є [0, 1].
$r_{\mathrm{off}}$	Value at random inside a certain range
$Y_{i}\left(t+1\right)$	ith individual’s location at iteration t+1
$Y_{best\;}\left(t\right)$	Best location at the current iteration
$Y_{M\;}\left(t\right)$	Mean locations of all individuals at the current iteration
$Y_{i}\left(t\right)$	Location of the ith individual at iteration t
L	Swarm population size
T	Maximum permitted iteration number
rand	Random number in a Gaussian distribution between 0 and 1
E	Dimensionality of the problems
Levy (E)	Levy flights
γ	Fixed value of 1.5
Γ	Gamma function
$Y_{R\;}\left(t\right)$	Randomly picked candidate
x and y	Spiral form
$r_{1}$	Fixed value ranging from 1 to 20
V	Constant 0.00464.
$E_{1}$	Array of integer numbers
ω	Constant 0.004
σ and ϵ	Two little fixed integers
$H_{1\;}\;$	Tracking motions
$H_{2\;}$	Flight slope of the AO
Q	Quality function
R	Maximum number of nodes.
$W_{r}$	Residual energy,
$W_{T}$	Total energy spent
$\;S_{\left(R\left(i\right)-B\right)}$	Distance between the ith node and BS
$S_{avg\left(R\left(i\right)-B\right)}$	Average distance between ith. node and BS
$S_{cr}$	Energy consumption rate
$S_{\left(c\left(i\right)\right)}$	Present location energy value of the ith node in the preceding round
$J_{i}$	Number of Sensor nodes
n(i)	Number of neighboring Sensor nodes
$\alpha_{1},\alpha_{2},\alpha_{3},\alpha_{4}\;\mathrm{and}\;\alpha_{5}$	Weighted values
$\alpha_{1},\alpha_{2},\alpha_{3},\alpha_{4}\;\mathrm{and}\;\alpha_{5}$.	0.33, 0.25, 0.20, 0.12 and 0.1
$A_{i}$	ith Aquila in the network
$SN\left(CH_{i}\right)$	Sensor node of ith CH
$P_{sSNBS}$	Separation between the SNs and BS
$RE_{SN}$	Residual energy of the subsequent SNs
